# Reduced CPU Workload for Human Pose Detection with the Aid of a Low-Resolution Infrared Array Sensor on Embedded Systems

**DOI:** 10.3390/s23239403

**Published:** 2023-11-25

**Authors:** Marcos G. Alves, Gen-Lang Chen, Xi Kang, Guang-Hui Song

**Affiliations:** School of Computing and Data Engineering, NingboTech University, Ningbo 315100, China; cgl@nit.zju.edu.cn (G.-L.C.); b20183080643@cau.edu.cn (X.K.); songnbt@nit.zju.edu.cn (G.-H.S.)

**Keywords:** edge computing, thermal image, MediaPipe, pose detection, power optimization, power saving

## Abstract

Modern embedded systems have achieved relatively high processing power. They can be used for edge computing and computer vision, where data are collected and processed locally, without the need for network communication for decision-making and data analysis purposes. Face detection, face recognition, and pose detection algorithms can be executed with acceptable performance on embedded systems and are used for home security and monitoring. However, popular machine learning frameworks, such as MediaPipe, require relatively high usage of CPU while running, even when idle with no subject in the scene. Combined with the still present false detections, this wastes CPU time, elevates the power consumption and overall system temperature, and generates unnecessary data. In this study, a low-cost low-resolution infrared thermal sensor array was used to control the execution of MediaPipe’s pose detection algorithm using single-board computers, which only runs when the thermal camera detects a possible subject in its field of view. A lightweight algorithm with several filtering layers was developed, which allowed the effective detection and isolation of a person in the thermal image. The resulting hybrid computer vision proved effective in reducing the average CPU workload, especially in environments with low activity, almost eliminating MediaPipe’s false detections, and reaching up to 30% power saving in the best-case scenario.

## 1. Introduction

Modern PC desktop systems, even using just an integrated graphics card, can run pose detection algorithms, such as MediaPipe [1], at around 10 to 15 frames per second (FPS), which is fast enough to give the impression of a fluid video stream. However, embedded systems have much more limited processing power and even less graphic performance. Considering applications that need real-time video/image processing, software and hardware must be optimized in all possible ways. An embedded system also has constraints from power consumption and maximum heat dissipation.

Several pose detection algorithms were proposed and are already available to use in the literature and the market such as YOLO, MediaPipe, OpenPose, DeepPose, PoseNet, and NVIDIA’s Body Pose Estimation, among others [1,2,3,4,5]. Most of them rely on the use of the GPU to boost performance and can achieve 25 FPS or more. As embedded systems for edge computing applications, such as the well-known Raspberry Pi and similar single-board computers (SBCs), have limited or no GPUs to be accessed by user applications, the MediaPipe framework is usually selected for SBCs since it can run with only the CPU for the detection algorithms with reasonable performance. New versions of MediaPipe can also use the GPU [6], but up to now, it has been limited to GPUs for conventional computers with x86_64 architecture, while GPUs at embedded architectures such as armv7 or aarch64 are not supported yet, at compilation or run time. There is support for mobile systems running Android or iOS, but they are out of the scope of this project. Nevertheless, it is possible for modern SBCs with GPUs that support OpenGL ES 3.1+ instructions to also benefit from hardware acceleration.

### 1.1. Related Work

With the advances in processing power on embedded systems, several applications using computer vision on this class of devices have been published in the last few years. For example, MediaPipe, which is widely used for machine learning, is an open-source platform maintained by Google [1]. In [7], a Raspberry Pi 4B with MediaPipe was used to recognize hand gestures with a camera and translate them into commands that can control other devices with infrared signals, replacing the remote control. In [8], a fall detection alarm system was developed using MediaPipe and Raspberry Pi 4B connected to a camera, achieving high accuracy in event detection. However, in [7,8], the MediaPipe algorithm must run all the time, always searching for a subject in the scene, even if there is not one. In [9], a relatively high-resolution thermal camera connected to a smartphone was used to determine blood alcohol concentration. The high resolution of a thermal camera allows for the application of more complex computer vision techniques in the analysis of the thermal image itself. However, this also leads to high CPU usage.

While MediaPipe is highly popular for computer vision applications, its face detection and, mostly, the pose detection algorithm require relatively high CPU usage and still exhibit false positives while tracking a possible person in the scene, depending on the video quality, illumination, and objects in the scene. False positives waste several computational resources, such as data storage, network traffic, CPU time, unnecessary power consumption, and consequently, high heat dissipation even when idle, with no person in the scene. Edge computing devices are especially impacted by the latter three cited items.

In this study, a new approach is proposed to optimize the computer resources used by MediaPipe’s pose landmark detection focused on embedded systems [10,11]. The MediaPipe pose detection task is controlled by a management program that decides when it can run based on information from an extra sensor. In brief, the management software receives additional data from a low-cost thermal array sensor and based on the detection of a person in the thermal image, controls the execution of the pose detection task. The detailed process is explained in Section 2.

## 2. Materials and Methods

This section describes the hardware and software used in the optimized pose detection system. A brief description of the chosen main computational platforms and sensors is provided. Microcontrollers and microprocessors such as STM32, STM32MP1, ESP32, and NXP, although enough for processing the thermal camera stage, are not capable of running the MediaPipe framework in terms of hardware and operational system. For the proposed task, single-board computers were chosen, as described in Section 2.1.

### 2.1. Test Platforms

#### 2.1.1. Single-Board Computers

The proposed solution was tested on five different SBCs and one minicomputer, the most advanced was an Orange Pi 5 (Orange Pi, Shenzhen, China), a cutting-edge SBC in the market (2022–2023) with an RK3588S processor running Armbian OS based on Ubuntu. The performance was compared with four popular SBCs on the market: a Raspberry Pi 4B (Raspberry Pi Foundation, Cambridge, UK), an Orange Pi 4 LTS (Orange Pi, Shenzhen, China), an NVIDIA Jetson Nano (NVIDIA, Santa Clara, CA, USA), and a minicomputer with an Intel N5105 mobile processor – Morefine N5105 (Morefine, Shenzhen, China). The Jetson Nano with 2 GB of RAM was chosen because it falls in the same price range as the Orange Pi 5 and the Raspberry Pi models, and the 4 GB model is also present for comparison. Still, all SBCs are considered low-cost equipment, and their prices were relatively similar at the time this article was written, although it is worth mentioning that the Orange Pi 5 has higher specifications and is cheaper than most of them. Table 1 summarizes the SBC specifications.

Different from the Raspberry Pi 4B, which is considered a general-purpose SBC, the Jetson Nano is aimed toward computer vision and other AI applications. The embedded GPU allows for the execution of several lightweight video processing applications with AI and machine learning algorithms. As shown in Section 3, the Jetson Nano can output an FPS three times higher than the Raspberry Pi 4B when running the standard pose detection algorithm from MediaPipe. The Orange Pi 4 LTS has built-in Flash Memory, and the Orange Pi 5 has an octa-core CPU and a slot for an NVMe SSD card, which was used for this project. The Jetson Nano and Raspberry Pi use a microSD card to store the operating system, which is much slower than the SSDs. Due to the Raspberry Pi’s high amount of RAM, this should not significantly impact the software performance while executing the developed solution. However, the Jetson Nano, with only 2 GB of RAM, is commonly considered inadequate for an SBC designed for computer vision applications; therefore, the 4 GB model was also included for comparison. The 2 GB of RAM limits the execution of multiple tasks and programs that require high amounts of memory. A clean boot in the 2 GB model already uses around 800 MB of RAM, leaving little memory for applications concerning computer vision.

It is also known that for the Jetson Nano, there are special SDKs designed to use the full potential of its GPU, such as NVIDIA TensorRT. However, for the sake of comparison, all SBCs were tested using MediaPipe’s framework. For the Raspberry Pi, the Armbian OS was also chosen for a fair comparison with the remaining SBCs.

The solution was developed entirely in the Python language, with Table 2 showing the Python version used for each test platform and the version of the main required packages. The Jetson Nano line is at the End-of-Life (EOL), and its operating system is not being updated anymore [12]. Therefore, it still ran Python 3.6, as an update for a newer version can raise many performance problems because of the optimizations made by NVIDIA. In addition, the standard OpenCV package native from NVIDIA’s official OS image is not compiled with CUDA optimizations for some unknown reason. To achieve better performance, the OpenCV was compiled from the source, which takes a considerable amount of time, enabling all the specific optimizations for the Jetson GPU. The MediaPipe package also must be compiled with CUDA support, and up to now, this was only possible with version 0.8.5.

Orange Pi 5, Orange Pi 4 LTS, and the Raspberry Pi 4B all ran the latest Armbian OS, which includes Python 3.10 and its packages. However, as some of the required packages are still unavailable for Python 3.10, Python 3.8 was chosen. The OpenCV available on the Armbian’s APT repository is already compiled with support for the NEON SIMD coprocessor embedded in the ARM family and can be directly used. The N5105 uses a desktop image of Ubuntu 22 and Windows 11, where no additional tinkering is needed.

#### 2.1.2. IR Array Sensor

**Figure 1:**
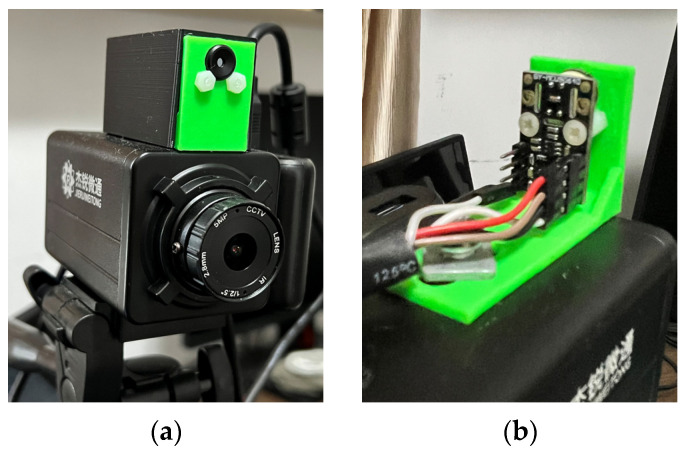
Mounted IR array sensor with camera. (**a**) visible light camera with the thermal camera mounted on the top. (**b**) Detailed view of the sensor inside the enclosure.

The core component that differentiates the proposed application is an infrared array sensor, or thermal camera (TC) as normally described. Unlike common image sensors—CCDs—the cost of TCs rises exponentially with the resolution. A thermal camera sensor with 256 × 256 pixels of resolution can cost more than the SBC itself. If the cost of all components combined is too high, there are better solutions instead of the addition of a thermal camera. With that said, a low-cost and low-resolution TC was selected for this application, achieving satisfactory results. The TC was made by Melexis (Melexis, Ypres, Belgium), model MLX90640BAA, with a 32 × 24 sensor array (768 pixels in total), 110-degree horizontal Field-of-View (FOV), and 75-degree vertical FOV. This sensor costs around USD 36.00, and the reconstructed thermal image is clear enough to discern human body features at close and medium distances, as can be seen in Section 3. Figure 1a shows the IR array sensor mounted on top of the camera utilized in the setup, Figure 1b shows a view of the sensor inside the box.

The MLX90640BAA has a configurable refresh rate between 0.5 Hz and 64 Hz, although, for practical applications, 16 Hz is the highest refresh rate due to the I2C maximum data rate in most SBCs (1 MHz), and 8 Hz is the highest when using the UART mode with a maximum baud rate of 460,800 bps. According to the manufacturer, a refresh rate higher than 8 Hz also introduces a considerable amount of noise in the thermal data. It is worth mentioning that the UART mode is not native to the sensor, and there is a dedicated microcontroller in the sensor’s board that makes the interface between I2C and UART. At the time this article was written, the I2C driver still had many issues when using the Armbian OS and consequently, the UART mode was selected with an 8 Hz refresh rate. This also allowed for testing the N5105 intel-based minicomputer.

Each data frame sent by the sensor contains an array with the temperature value of each individual pixel in Celsius degrees, 768 in total, plus some other additional data. The complete data frame when working in UART mode can be seen in Table 3.

#### 2.1.3. Main Camera IMX219 with a 120-Degree Field-of-View

The main camera consists of an IMX219 1080p@30FPS with 8MP of resolution and a 120-degree diagonal FOV, a 105-degree horizontal FOV, and a 59-degree vertical FOV. The wide angle of the FOV is an important parameter, as it allows an almost compatible image with the one provided by the thermal camera from Melexis. In other words, both the main camera and the thermal camera can observe the same area.

### 2.2. Pose Detection Aided by the Thermal Camera—PDATC

**Figure 2:**
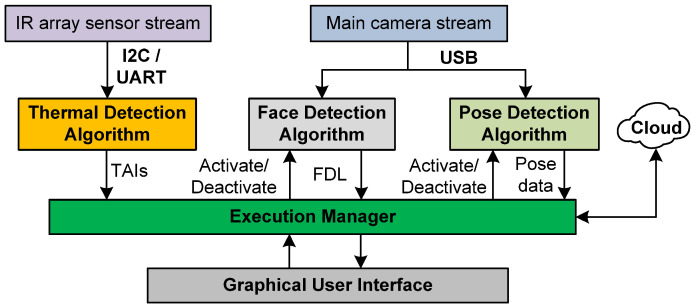
Overview of the proposed system—PDATC.

Figure 2 shows the main structure of the developed software. PDATC (1.0b), made entirely in the Python language, is divided into seven main threads that govern the application: the execution manager (EM), the thermal detection algorithm (TDA), the face detection algorithm (FDA), the pose detection algorithm (PDA), the graphical user interface (GUI), and two more threads dedicated to providing data and video streams from the thermal camera and the visible light camera.

The EM governs the state machine that controls the algorithms for person detection. The GUI is an independent thread, pulling data from the EM. The program life cycle goes as follows: initially, the EM only runs the TDA. The thermal camera is used as the primary activating agent for the face detection and pose detection algorithm, which remains idle when the TDA cannot detect a subject in the scene. Once the TDA detects a possible subject, the 2D boundaries (height and width) are determined and stored with the name TAI—thermal area of interest—where one or more TAIs are stored in a data list. If a TAI is received by the EM, it activates the FDA, which will search for a person’s face in the image from the main camera for up to three seconds. When one or more faces are detected, the FDA stores their 2D boundaries and returns a face detection list (FDL) to the EM.

**Figure 3:**
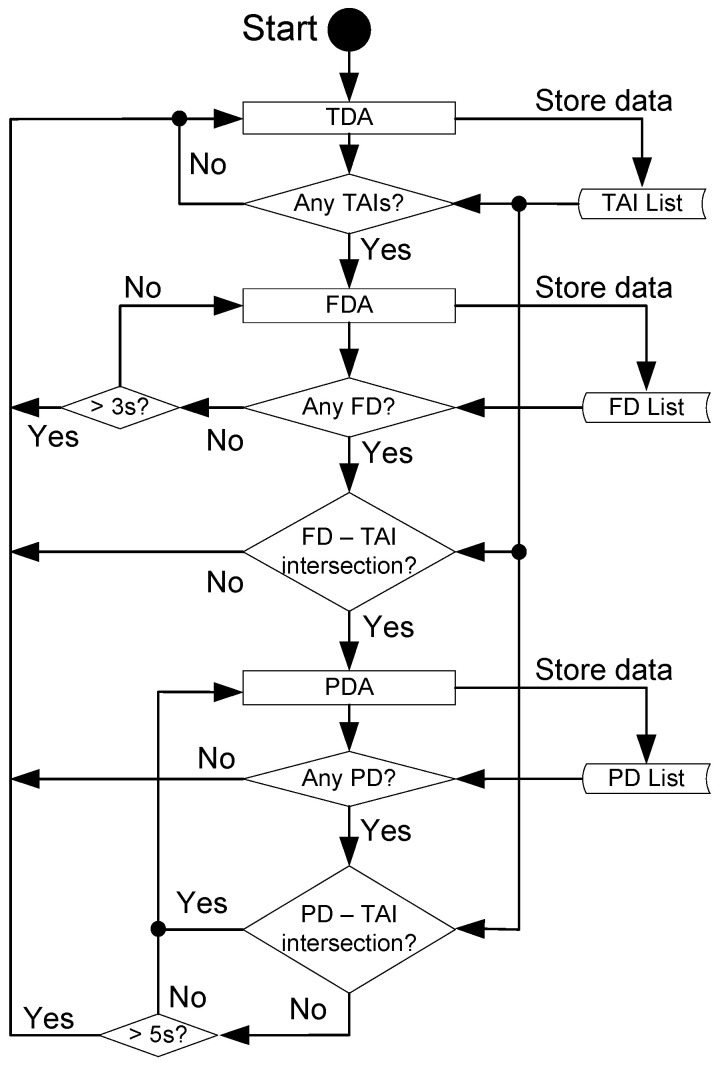
PDATC logic flow diagram.

At this point, the EM compares the area location of each item in the FDL with the area location of the TAIs. If they do not intersect, it is considered a false positive and ignored, and the EM goes back to the TDA. When a face detection area location intersects a TAI, the EM deactivates the FDA and activates the PDA to track the person’s pose. The PDA returns the pose data, with the body segment position and the 2D boundaries of the entire body. The area location of the pose data in the image is constantly compared with the list of TAIs, checking for an intersection. If there is no intersection between the pose data and any TAI for five seconds, the EM deactivates the PDA and goes back to the TDA. Figure 3 shows the flow diagram of the PDATC.

This method proved effective in removing MediaPipe’s false positives, as well as deactivating the demanding pose detection algorithm when it is not necessary. The cloud connection depicted in Figure 2 illustrates the possibility of sending the detection data to a cloud database or sending other information, but the program does not need an internet connection to operate and can store data locally.

#### 2.2.1. Thermal Detection Algorithm—TDA

To determine the TAI, the thermal detection algorithm works with the raw data from the IR array sensor, as the number of points to go through will be much lower than the constructed and up-scaled thermal image. Several layers of decisions are used to validate a TAI as a possible person.

First, the TDA searches the IR raw data matrix for thermal points with a temperature in the range of the human body, considering the standard skin temperature between 26 °C and 40 °C (named Tmin and Tmax) [13]. The X–Y coordinate of each thermal point in this range is then added to a separate data list. During the second step, a Grassfire-based algorithm is used to join the data points in the list that are neighbors, forming larger groups [14]. This process continues until all X–Y data points are covered. The minimum and maximum X–Y coordinates in each group are used to form the 2D boundary that delimits the TAI. A third step removes all the TAIs with fewer than 20 points of thermal data, with the intention to discard small areas in the TC FOV that cannot fit a person. A minimum of 20 points from the thermal data was determined using two parameters. First, it was considered that the subject is positioned two meters from the cameras. Using the horizontal and vertical FOV of the TC, each pixel in the thermal camera at this distance represents ~17.9 cm horizontal span and ~8.7 cm vertical span, with an area of approximately ~155 cm^2^. Second, using [15] as a standard, the average 2D area of the human body positioned facing the camera was considered to be around 5583 cm^2^, considering height and interscye breadth as parameters. With that, using 20 points of the TC data at this distance removes objects with an area smaller than half of an average human body, around ~3100 cm^2^.

With these three steps, areas of interest in the thermal image can be isolated. However, to improve the ability to detect a person in a low-resolution thermal image, a fourth filter is included. The standard model of a human body heat distribution follows a Gaussian distribution or normal distribution [13]. Taking advantage of this, an algorithm based on D’Agostino and Pearson’s is used to check the null hypothesis of the thermal data in each TAI to verify if it fits a normal distribution [16,17,18]. This test combines skew and kurtosis to produce a test of normality. The function returns two values: the first is a statistic value based on the skew test squared plus the kurtosis test squared and the second is a 2-sided chi-squared probability for the hypothesis test, called the *p*-value. Only the *p*-value is used for the decision-making process. If the thermal data do not come from a normal distribution, the *p*-value will have a very small value of less than 1.0 × 10^−3^ and typically less than 1.0 × 10^−10^. An alpha constant (α) was defined as 1.0 × 10^−5^ to be considered the boundary for a normal distribution, so if the TAI receives a score greater than alpha, it is considered to enter the TAI list.

**Figure 4:**
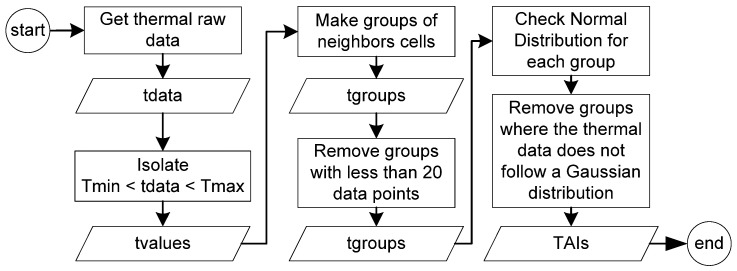
Thermal data analysis algorithm. From raw thermal data to the TAI list.

After the filtering process, each remaining TAI contains the X–Y boundary box, time of creation, time of the last *p*-value > α, unique ID, normal distribution score (*p*-value), and thermal data. Every iteration of the TDA revalidates each TAI, giving them a new *p*-value score and updating the time of the last *p*-value > α while keeping the ID and time of creation. For a TAI to be allowed to enter the TAIs list, it must receive a *p*-value greater than alpha for more than three consecutive seconds; otherwise, it will be discarded and not available for the EM. After that, if the *p*-value for a given TAI in the TAI list becomes lower than alpha, the TAI will “live” for up to five consecutive seconds until it receives a *p*-value greater than alpha; otherwise, it will be discarded. As a common situation, there will be times when the X–Y boundary boxes of two or more TAIs intersect each other, such as when more than one person is in the scene. In this case, if the thermal data points in the range of interest do not overlap, each TAI will be treated as an individual. If the thermal data points in the range of interest of two TAIs overlap, these TAIs will be merged in the “make groups of neighbors cells” stage, shown in Figure 4, becoming one large TAI with only one unique ID.

Finally, the TAI list is available for the EM, which receives all TAIs that were confirmed as a valid thermal area of interest. Figure 4 shows the process from the raw thermal data until it becomes a TAI. tdata, tvalues, and tgroups are all lists, where tdata contains the raw data from the sensor, tvalues contains only the data that fits between Tmin and Tmax, and tgroups is a list with groups formed by the grouping step.

Considering a typical home environment with sources of heat signatures commonly present such as pets, heaters, air conditioning systems, electronic devices, and small hot objects (foods and beverages), several procedures to isolate a TAI guarantee almost no false positives. Pets and small objects with heat signatures are removed in the stages “Isolate Tmin < tdata < Tmax” and/or “Remove groups with less than 20 data points”, as shown in Figure 4. Large objects such as heaters and TVs are discarded with “Isolate Tmin < tdata < Tmax” and/or by receiving a *p*-value less than alpha. If any false positive TAI is still present and becomes part of the TAI list, the FDA stage will most likely block it from activating the PDA, as explained in Section 2.2.2.

The search range of the temperature of interest, Tmin and Tmax, was determined as follows: the maximum temperature Tmax was defined as the maximum normal temperature for a human body, considering a fever from a common cold (40 °C) [19]. The minimum temperature Tmin, however, depended on many factors, such as the subject’s distance from the TC and the ambient temperature. Due to the thermal camera’s low resolution, the heat from the body of a subject at medium distances, more than 3 m away from the TC, tends to spread out and cause the thermal readings to be lower than the real value. In other words, each TC pixel has the average temperature of a larger area, as the more distant a subject is from the TC. For person detection, this reading error tends to get worse as the ambient temperature (Ta) gets lower. On the other hand, if the ambient temperature is elevated (>32 °C), it becomes difficult to discern a human body from the surrounding area. In brief, a static Tmin will work only for a specific ambient temperature. In this study, Tmin varies dynamically with the ambient temperature, defined as Equation (1). The ambient temperature Ta can be obtained from the thermal camera data (Table 3).

Tmin = Ta + Toffset(1)

where Toffset is defined as 1.5 °C, pushing Tmin higher than the ambient temperature. This approach guarantees that the minimum temperature does not match Ta, which will render the TDA ineffective in the grouping step due to the high amount of data in the range of interest. Also, it is known that the proposed system will not work properly when Ta is near normal human body temperature. In this case, the TDA is disabled, and the EM works only with the FDA to control the execution of the pose detection algorithm.

#### 2.2.2. Face Detection Algorithm—FDA

In this study, the face detection from the MediaPipe framework is used as an auxiliary tool for the TDA. After the execution manager confirms one or more valid TAIs, it will activate the face detection algorithm, which is dormant until this point.

The FDA will try to detect a face in the video stream for up to three seconds, and if successfully detected, it stores the face’s X–Y boundaries given by MediaPipe. The EM then can check if there is any area intersection with a TAI. If an intersection is positive, the FDA is deactivated, and the EM moves to the pose detection algorithm.

#### 2.2.3. Pose Detection Algorithm—PDA

Pose detection in this project uses the MediaPipe framework pose detection algorithm, which uses BlazePose 33 landmark topology, a superset of COCO key points, Blaze Palm, and Blaze Face topology, and can track one person in the scene at time [20]. One can choose three models for pose estimation: BlazePose GHUM Heavy, BlazePose GHUM Full, and BlazePose GHUM Lite. The Heavy model is indicated for high-end computers, due to its complexity, and presents high accuracy; the Full model balances accuracy and workload; the Lite model is indicated for low-resource computational systems, although the accuracy is considerably compromised. In this study, the BlazePose GHUM Full model is selected since the set of SBCs used for testing can run the Full model with acceptable performance.

MediaPipe’s face detection and pose detection solutions were not modified by any means in this work, except that the execution manager decides when it can activate and deactivate its execution. After an intersection between a TAI and an FDA is confirmed by the EM, the PDA is activated and starts tracking the person’s pose. When there is no intersection between the pose data and a TAI, the EM deactivates the PDA and goes back to the thermal detection algorithm stage.

As pose detection algorithms heavily use the CPU, even when there is no person in the scene, an external way to control when it can be executed proves to be highly beneficial for embedded systems. Section 3 shows the comparative results with and without the aid of PDATC.

## 3. Results

This section presents the results obtained with the proposed system. The PDATC-developed software main screen and detection results are shown. Then, a comparison of the algorithms FPS, power consumption, CPU usage, and temperature between the pose detection alone and with the PDATC solution among all tested SBCs are shown.

### 3.1. PDATC Software Detection Results

**Figure 5:**
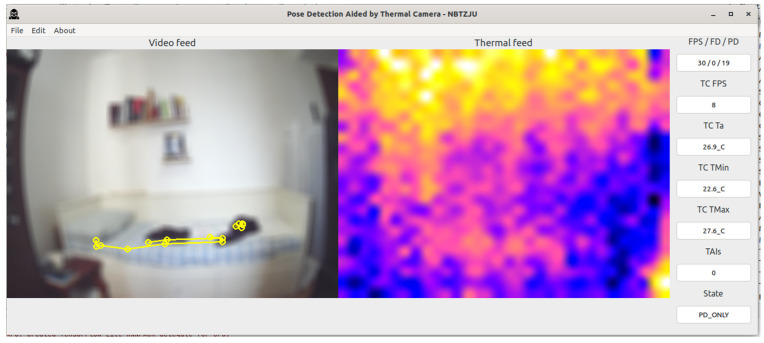
Only MediaPipe’s pose detection running with false a positive detected (body segmentation in yellow on the image from the visible camera).

**Figure 6:**
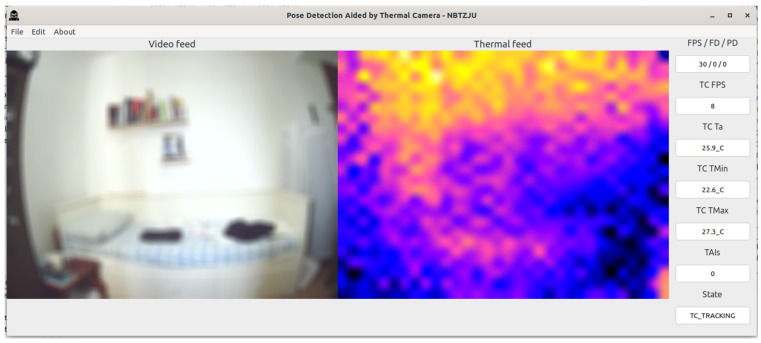
PDATC activated with no subject in the scene.

**Figure 7:**
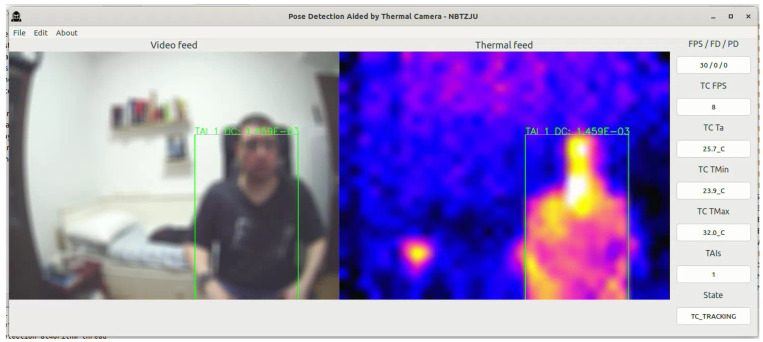
PDATC active with a subject entering the scene. The green box on the right shows the location of a TAI. The coordinates are transferred to the visible light camera on the left.

**Figure 8:**
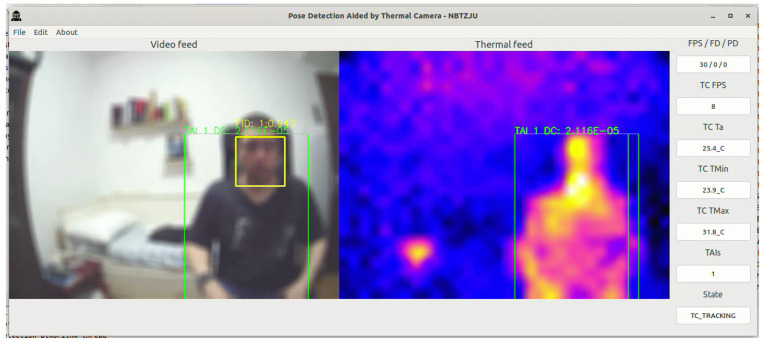
PDATC active with the face detection stage operating. The green box shows the location of a TAI. The yellow box shows the face detected by the FDA.

**Figure 9:**
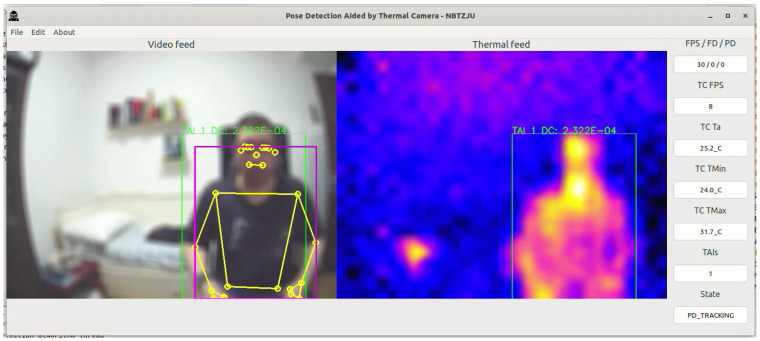
PDATC active with pose detection running. The green box shows the location of a TAI. The purple box shows the area where a person is detected by the PDA and the body segmentation is shown in yellow on the image from the visible camera.

**Figure 10:**
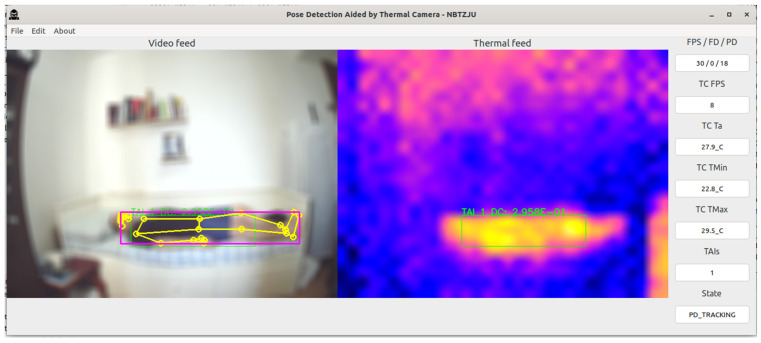
PDATC active with a subject on the bed. The green box shows the location of a TAI. The purple box shows the area where a person is detected by the PDA and the body segmentation is shown in yellow on the image from the visible camera.

**Figure 11:**
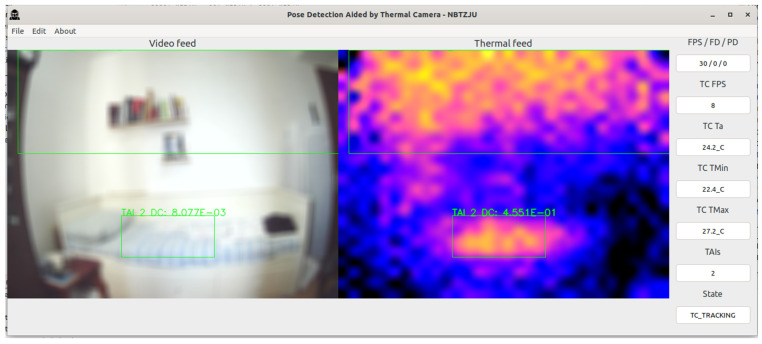
PDATC active after the subject leaves the scene. The green box shows the location of a TAI.

**Figure 12:**
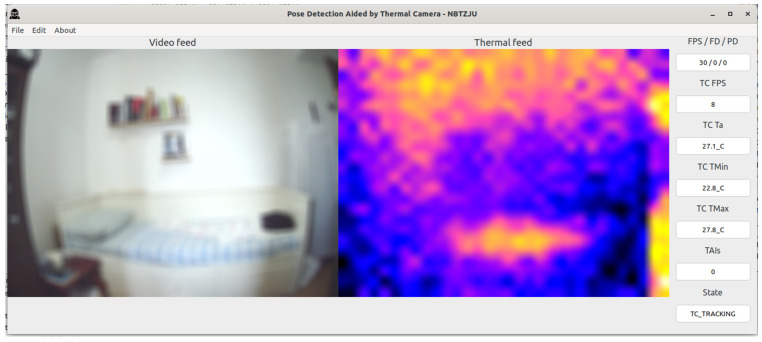
PDATC active after the subject leaves the bed. TAIs invalidated and discarded.

The PDATC-developed software main screen, obtained from the Orange Pi 5, can be seen in Figure 5, Figure 6, Figure 7, Figure 8, Figure 9, Figure 10, Figure 11 and Figure 12. Using Figure 5 for the description, there are two video feeds: the first one on the left comes from the normal camera, named “video feed”, and on the right is the video stream from the thermal camera, named “thermal feed”. There is also a column in the rightmost part of the screen, showing several data points and the status of the software.

The video feed is collected and processed internally using a resolution of 1920 × 1080p @30FPS and a thermal feed with 32 × 24p @8FPS. The video feed is downscaled to 640 × 480p and the thermal feed is upscaled to 640 × 480p using cubic interpolation for better image quality. The resizing of both feeds is only for presentation on the screen; the data are used at the original resolution by the algorithms. Also, the blurring in the video feed is on purpose for publication privacy, and only for visualization on the screen. For the data column, the important fields are the first one (FPS/FD/PD), indicating the video feed, the FDA, and the PDA FPS; the TAI field, with the number of detected TAIs; and the last one (State), indicating the status of the PDATC.

Figure 5 shows the test environment used for data collection, assuming a hypothetical case where a person is monitored while in the bedroom for safety purposes. In this figure, there is no subject in the scene and the PDATC software is configured to run only MediaPipe’s pose detection (State: PD_ONLY). It is possible to notice that MediaPipe detects a false positive occurrence of a person in the scene, increasing the CPU usage and generating false data. The minimum detection confidence (MDC) was set to 0.85 in the pose detection algorithm and 0.75 as the minimum tracking confidence (MTC). An MDC and MTC too close to 1.00, although drastically reducing the occurrence of false positives, leads to intermittent detections and delays in detecting the subject, and the pose detection algorithm itself will still run all the time.

In Figure 6, the PDATC solution is active. There is no subject in the scene, as can be seen in the thermal feed, and no TAI is detected with the TDA. In this state, the FDA and PDA algorithms are on pause, freeing up computational resources for other tasks and to save energy.

When a subject enters the scene, as can be seen in Figure 7, the TDA tracks and isolates the boundary box around the subject, following the procedure described in Figure 4. For demonstration purposes, there is also a hot teacup in the same scene, which is properly filtered with the TDA and not recognized as a TAI.

After a TAI is received, the FDA is activated and confirms if there is a person’s face inside the same 2D boundary box of the TAI (Figure 8). After the confirmation, pose detection can start, as shown in Figure 9.

In Figure 10, the subject is resting on the bed and the PDATC keeps tracking the person in the thermal feed without problems. Observing Figure 11, when the subject leaves the scene, there is still a thermal footprint on the bed, which the TDA initially indicates as a TAI. This TAI is later invalidated and discarded using either the TDA or the FDA (Figure 12). To avoid overusing the FDA in the case of a false positive TAI, the area of each discarded TAI remains invalid for 1 min before it can be analyzed again with the FDA.

### 3.2. SBC Performance Regarding the Algorithms’ FPS

Table 4 shows the average FPS for each SBC regarding the main algorithms. All SBCs were able to sustain a video feed of 30 FPS except for the Raspberry Pi 4B, which needs to be configured to 15 FPS due to performance restrictions. The thermal camera reached 8 FPS on every SBC, and the FDA was configured to a maximum of 15 FPS because there is no need for a higher FPS on this application. There was no FPS upper limit imposed on the PDA algorithm. The Orange Pi 4 LTS only reached 13 FPS for the FDA, and the Raspberry Pi 4B was able to reach 9 FPS, showing the limitations of these two SBCs. The Raspberry Pi also has poor performance of the pose detection algorithm, with an average of 5 FPS while tracking a person.

### 3.3. CPU Utilization and Temperature

While pose detection is on pause by the PDATC, the CPU utilization is considerably lower since the TDA algorithm requires less CPU time. Table 5 shows the CPU average usage, considering all cores, and temperature with only MediaPipe’s pose detection and the PDATC activated. Table 5 also shows a comparison of the CPU SOC temperature in the same conditions. The data were collected using an average 15 min window, and the temperature is the final temperature after the test. The test environment is the same as shown in Figure 6, with no subject present in the scene during the duration of the test. While running only the pose detection algorithm, there were a few false positives detected, but the sum of the total time tracking them was less than 30 s on average for all SBCs. Considering the sampling window of 15 min, they were not included in Table 5. With the PDATC active, there were no false positives detected; thus, the pose detection algorithm was not activated at any moment. It is important to highlight that even without false positives, pose detection alone considerably elevates CPU usage. The environment with no subject in the scene was chosen because it will result in the best-case scenario for the PDATC, leading to the lowest CPU usage and highest power saving. With a subject in the scene, pose detection will be always activated, resulting in the same CPU usage and power consumption as the case running only pose detection. As the objective of PDATC is to deactivate pose detection when there is no person in the scene, Table 5 presents the comparison with and without PDATC for this scenario.

It is also possible to note a significant drop in the temperature of the SBC’s board working temperature. Table 6 shows the overall temperature distribution in all SBCs with only MediaPipe’s pose detection and with PDATC. All SBCs were equipped with the recommended heatsinks and fans. The ambient temperature was controlled at 25 °C, and the SBC body average temperature was measured with the thermal camera software, where you can select only the SBC body for calculation.

### 3.4. Power Consumption

Another goal of the PDATC solution is to reduce power consumption while there is no subject in the scene (by reducing CPU utilization). Table 7 shows the results overview for the power consumption among all tested computers for the idle state (only the operating system), with only pose detection running and with PDATC activated. For this scenario, the SBCs were disconnected from the internet, with no WiFi, ethernet, or Bluetooth connection. The results were collected using a USB power analyzer connected to the SBC power supply input. Due to the high variability in power consumption in computation systems, each result is an average of a 10-min window with a sample rate of 20 Hz.

In the last column of Table 7, it is worth noting that all SBCs presented a considerable reduction in power consumption while using the PDATC solution compared with only pose detection. The Orange Pi 5 and 4 and the N5105 running Ubuntu reached around 30% power savings.

## 4. Discussion

As seen in Table 7, the average power consumption considering all tested SBCs was reduced by 23.03%, reaching an economy of 29.12% in the Orange Pi 5. The Raspberry Pi 4B benefited less from the inclusion of the PDATC; however, its performance for pose detection is already low. Still concerning the Raspberry Pi 4B, it is worth mentioning that the BlazePose GHUM Lite model was also tested on this SBC, reaching 7 FPS with the subject in the scene, but as the accuracy was considerably reduced, we decided to not include the comparison using the Lite model on this article.

Table 4 shows that the Orange Pi 5 surpasses the performance of the Jetson Nano while consuming less power and running cooler. The Orange Pi 5 is considerably newer and more technologically advanced, and with an octa-core SOC, does a better job running multitasking applications. The Orange Pi 4 LTS has a hexa-core CPU, which also gives it an edge compared with the Raspberry Pi 4B and the Jetson Nano in this scenario.

The Jetson Nano’s oddly low performance, considering that it has an embedded CUDA GPU, is mostly due to software limitations. As stated, the Jetson Nano has specific SDKs for image processing and, although MediaPipe and OpenCV were compiled with CUDA support, they cannot take full advantage of the GPU as the NVIDIA SDK could. Therefore, most tasks are still run on the CPU, which has lower performance compared with the other SBCs. Regardless, the Jetson Nano is considerably expensive and is at its EOL, so it is not recommended for new projects. It is also possible to note that the 4 GB model presents higher CPU usage in the results. This model uses a different operating system image than the 2 GB, which uses a highly optimized Linux version due to RAM limitations.

It is worth pointing out that for this application, there are other solutions that can be used to activate/deactivate the pose detection algorithm. For example, a radar system can be applied, like the mmWave line from Infineon, ultrasonic sensors, or simpler motion detection sensors. However, the data and applications supplied by a thermal camera can surpass the usability of other types of sensors in several ways. In contrast to the thermal camera, most of the common motion sensors need to be mounted statically to be accurate and will detect any motion, including objects. More advanced radar sensors are too expensive for the scope of this project. In addition, the thermal camera can provide an additional video stream where there is no need for privacy concerns.

False positive occurrences in MediaPipe are becoming lower with every new version, but the required computational resources are becoming higher, with the newest version 0.10.3 being able to take more advantage of the GPU as well. For edge computing, it is interesting to keep the workload CPU bounded due to the characteristics of this class of devices. Although it is still possible that a false positive detected with the TDA/FDA will activate pose detection, the constant revalidation of the subject limits the time when a false positive is tracked. The fine-tuning of the thermal detection algorithm is a critical component, which can lead to intermittent activation/deactivation of pose detection by the TDA, or no detection at all, if not performed correctly. The confirmation times mentioned in Section 2.2.1 to validate and invalidate a TAI can also be adjusted for faster detection of the subject, although the presented values achieved good results in avoiding false positives.

It is known that the TDA algorithm needs to be tested on more varieties of scenarios in which complex situations may cause the detection of false positives, elevating the usage of the face detection algorithm to validate the TAI. For future work, there is an enhanced version of the TDA algorithm for human detection based on the low-resolution thermal image currently in development. The new version is being developed using a low-complexity neural network to not compromise CPU usage. There are still false positive detections of TAIs with the current algorithm, hence the use of face detection as the last filter to confirm the TAI validity. The present solution, although strictly procedural, achieved good results with low-resolution thermal images; however, there is room for improvement while keeping this algorithm as light as possible.

## 5. Conclusions

The addition of a low-cost IR array sensor to control the activation of the pose detection algorithm led to a more efficient use of the limited computational resources on embedded systems. The controlled activation and deactivation of the pose detection algorithm and the near elimination of false positives create a more efficient idle state when there is no person in the scene, reducing the CPU utilization and generation of unnecessary thermal heat and data. The thermal detection algorithm, being relatively simple and strictly procedural, requires little CPU usage due to the low resolution of the thermal camera. The PDATC proposed solution will achieve the highest yield in environments with low human activity, where the pose detection algorithm can stay deactivated most of the time. As described in Section 1.1, there are several applications using computer vision on embedded systems, and they can benefit from the smart activation and deactivation of the demanding CPU usage that this class of application normally presents.

It is important to highlight that the addition of a thermal camera can bring other positive points. For example, thermal cameras do not require an external light source to operate, and they can be used to activate lights when a possible person is detected in the scene during poor light conditions, where a common camera will probably fail. In this solution, the execution manager can also be used to trigger other tasks while the system is running in thermal detection mode, for example, uploading the necessary data to a cloud service, leading to a more even CPU utilization.

The benefits of combining different types of sensors for computer vision, with different wavelengths, proved highly beneficial while keeping a low cost. This hybrid setup can be easily extrapolated and customized for other applications. The reduction in overall power consumption and CPU temperature is significant when there is no person in the scene, which leads to a more stable operation and improves the lifetime of the embedded system.

## Figures and Tables

**Table 1:** SBC test platforms’ specifications.

SBC	CPU	GPU	RAM	Storage	OS
Orange Pi 5	RK3588S octa-core (Quad-Core Cortex-A76 @ 2.4 GHz and Quad-Core Cortex-A55 @ 1.8 GHz)	Arm Mali-G610 MP4 “Odin”	8 GB	NVMe 256 GB	Armbian OS—Ubuntu 22.04
Orange Pi 4 LTS	Rockchip RK3399 (Dual-Core Cortex-A72 @ 1.8 GHz and Quad-Core Cortex™-A53 @ 1.8 GHz)	Arm Mali-T860	4 GB	eMMC 16 GB	Armbian OS—Ubuntu 22.04
Raspberry Pi 4B	Broadcom BCM2711 Quad core Cortex-A72 (ARM v8) 64-bit SoC @ 1.8 GHz	Broadcom VideoCore VI	8 GB	microSD card Class 10 64 GB	Armbian OS—Ubuntu 22.04
Nvidia Jetson Nano 2G	Quad-core ARM A57 @ 1.43 GHz	128-core NVIDIA Maxwell™ architecture-based GPU (accessible to user applications)	2 GB	microSD card Class 10 64 GB	JetPack 4.6.3 Ubuntu based distribution
Nvidia Jetson Nano 4G	Quad-core ARM A57 @ 1.43 GHz	128-core NVIDIA Maxwell™ architecture-based GPU (accessible to user applications)	4 GB	USB3.0 SSD 256 GB	JetPack 4.6.3 Ubuntu based distribution
MiniPC Morefine N5105	Intel^®^ Celeron^®^ N5105—Mobile Series Quad-Core @ 2.0 GHz	Intel^®^ UHD Graphics	8 GB	NVMe 256 GB	Ubuntu 22.04
MiniPC Morefine N5105	Intel^®^ Celeron^®^ N5105—Mobile Series Quad-Core @ 2.0 GHz	Intel^®^ UHD Graphics	8 GB	NVMe 256 GB	Windows 11

**Table 2:** Python and the main Python package versions used in this project.

SBC	Python	MediaPipe	wxPython	Scipy	OpenCV	Numpy
Orange Pi 5	Python 3.8	0.10.1	4.2.0	1.10.1	4.7.0	1.24.2
Orange Pi 4 LTS	Python 3.8	0.10.1	4.2.0	1.10.1	4.7.0	1.24.2
Raspberry Pi 4B	Python 3.8	0.10.1	4.2.0	1.10.1	4.6.0	1.24.2
Jetson Nano 2G	Python 3.6	0.8.5-cuda102	4.1.0	1.5.4	4.6.0-cuda102	1.19.4
Jetson Nano 4G	Python 3.6	0.8.5-cuda102	4.1.0	1.5.4	4.7.0-cuda102	1.19.4
Morefine N5105 Ubuntu	Python 3.8	0.10.1	4.2.0	1.10.1	4.7.0	1.24.2
Morefine N5105 Windows	Python 3.8	0.10.1	4.2.0	1.10.1	4.7.0	1.24.2

**Table 3:** Data frame from the MLX90640BAA thermal sensor in UART mode.

Byte	0–1	2–3	4–1539	1540–1541	1542–1543
Information	Frame header	Data length	Temperature data	Sensor/ambient temperature	Checksum16

**Table 4:** FPS for each SBC and main algorithm.

SBC		Normal Camera	Thermal Camera	Face Detection	Pose Detection (without Subject)	Pose Detection (with Subject)
	FPS					
Orange Pi 5		30	8	15	23	19
Orange Pi 4 LTS		30	8	13	9	7
Raspberry Pi 4B		15	8	9	7	5
Jetson Nano 2G		30	8	15	14	13
Jetson Nano 4G		30	8	15	15	13
N5105 Ubuntu		30	8	15	13	11
N5105 Windows		30	8	15	13	10

**Table 5:** CPU average usage, considering all cores, and CPU temperature.

SBC	CPU Average Usage with Only Pose Detection (%)	CPU Average Usage with PDATC (%)	CPU Temperature with Only Pose Detection (°C)	CPU Temperature with PDATC (°C)
Orange Pi 5	32.0	18.2	59.2	42.5
Orange Pi 4 LTS	45.3	22.4	83.9	58.9
Raspberry Pi 4B	63.8	32.2	59.2	51.6
Jetson Nano 2G	42.3	36.5	40.0	36.5
Jetson Nano 4G	51.4	43.9	43.5	41.5
N5105 Ubuntu	35.1	21.7	58.0	42.0
N5105 Windows	45.4	20.2	60.6	43.3

**Table 6:** Temperature distribution with only MediaPipe’s pose detection and with PDATC.

#	SBC	With Only Pose Detection	With PDATC
1	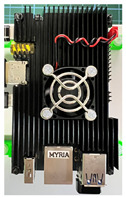 (**a**) Orange Pi 5	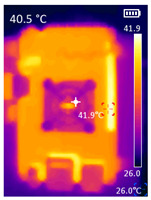 (**b**) SBC body average: 41.1 °C	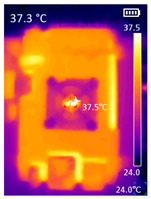 (**c**) SBC body average: 36. 3°C
2	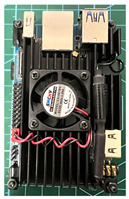 (**a**) Orange Pi 4 LTS	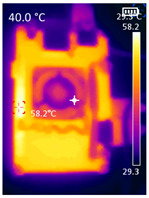 (**b**) SBC body average: 47.0 °C	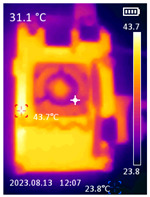 (**c**) SBC body average: 36.1 °C
3	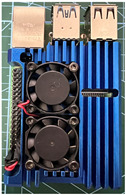 (**a**) Raspberry Pi 4B	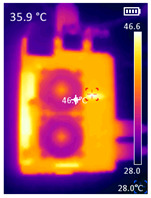 (**b**) SBC body average: 39.6 °C	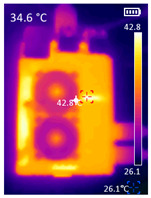 (**c**) SBC body average: 36.6 °C
4	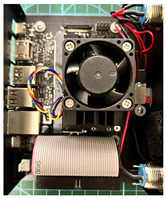 (**a**) Jetson Nano 2G	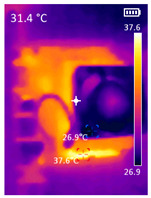 (**b**) SBC body average: 32.6 °C	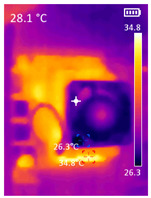 (**c**) SBC body average: 30.6 °C
5	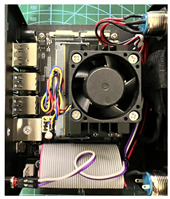 (**a**) Jetson Nano 4G	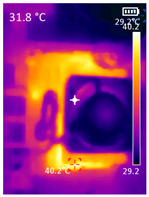 (**b**) SBC body average: 33.7 °C	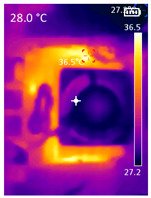 (**c**) SBC body average: 30.9 °C
6	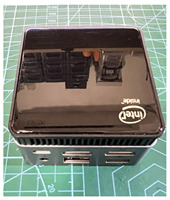 (**a**) N5105 Ubuntu	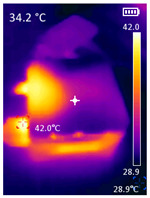 (**b**) SBC body average: 34.6 °C	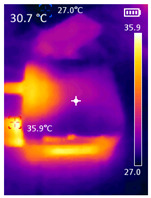 (**c**) SBC body average: 31.2 °C
7	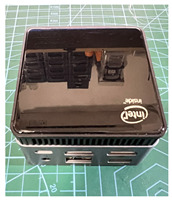 (**a**) N5105 Windows 11	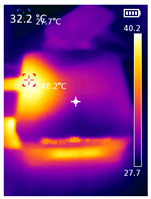 (**b**) SBC body average: 33.2 °C	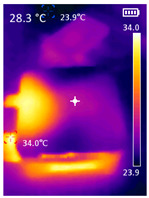 (**c**) SBC body average: 28.5 °C

**Table 7:** SBC power consumption results.

SBC		Idle	Only MP Pose Detection	with PDATC	Reduction with PDATC	Relative Power with PDATC (%)
	Power (W)					
Orange Pi 5		3.72	6.99	4.96	2.04	−29.12
Orange Pi 4 LTS		3.43	8.21	5.70	2.51	−30.54
Raspberry Pi 4B		3.75	6.27	5.65	0.62	−9.96
Jetson Nano 2G		3.38	7.21	5.42	1.79	−24.81
Jetson Nano 4G		4.89	8.86	7.11	1.75	−19.79
N5105 Ubuntu		6.38	15.02	10.63	4.93	−29.23
N5105 Windows		5.62	14.34	11.79	2.55	−17.76

## Data Availability

Data are contained within the article.
